# Root- and foliar-applied silicon modifies C: N: P ratio and increases the nutritional efficiency of pre-sprouted sugarcane seedlings under water deficit

**DOI:** 10.1371/journal.pone.0240847

**Published:** 2020-10-15

**Authors:** Gelza Carliane Marques Teixeira, Renato de Mello Prado, Antonio Márcio Souza Rocha, Marisa de Cássia Piccolo

**Affiliations:** 1 Laboratory of Plant Nutrition, Department of Soils and Fertilizers, São Paulo State University (UNESP), Jaboticabal, São Paulo, Brazil; 2 Laboratory of Biogeochemistry, Department of Technology, São Paulo State University (UNESP), Jaboticabal, São Paulo, Brazil; 3 Laboratory of Nutrient Cycling, Center of Nuclear Energy in Agriculture (CENA), University of São Paulo (USP), Piracicaba, São Paulo, Brazil; Harran University, TURKEY

## Abstract

Water deficit limits the establishment of sugarcane plants from pre-sprouted seedlings (PSS). Silicon (Si) can mitigate such stress, and your supply in plants with the active absorption mechanism is carried out through roots. However, foliar spraying has been practiced to supply Si in PSS production nurseries. Althought it is known that Si via roots can alter C: N: P ratios, nothing has been reported about its supply via foliar spraying, nor whether such changes interfere with structural nutrient use efficiency and with plant physiological responses. Thus, this study aimes to asses whether Si foliar spraying changes C: N: P ratios and increases the nutritional efficiency of PSS, as well as whether water deficiency interferes with such a relationships. For these purposes, three experiments were carried out. In experiment I, treatments consisted of two sugarcane cultivars (CTC 4 and RB 966928) and three Si supply forms (in nutrient solution via roots [SiR], via foliar spraying [SiL], and one control with the absence of Si [-Si]). The same Si supply forms were used in the other two experiments. In experiment II, a short-term water deficit was induced by polyethylene glycol addition to nutrient solution (-0.6 MPa) for three days. In experiment III, a long-term water deficit was imposed using levels of soil water retention capacity (70% [no water deficit], 50% [moderate water deficit], and 30% [severe water deficit] for 30 days. Our findings revealed that Si supply decreased C concentrations regardless of water conditions and that N and P concentrations varied with Si supply form and water deficit level. Moreover, root- and foliar-applied Si modified the C: N: P stoichiometry and increased C use efficiency in PSS, which thus increased N and P use efficiencies. Such an increased nutritional performance helped adjust physiological parameters and increase dry matter yield in PSS, both under water stress and non-stress conditions. Further, Si foliar spraying promoted structural effects on PSS regardless of water conditions, even if sugarcane has an active absorption via roots. In conclusion, foliar spraying can be used to supply Si in PSS production nurseries.

## 1. Introduction

Sugarcane global production has increased significantly due to its importance as a renewable energy source [1]. Pre-sprouted seedling (PSS) production has currently been used in sugarcane plantations. It consists of individualizing stems for emergence before transplanting [2]. This seedling production system is one of the biggest innovations in the sugar and alcohol sector, as it allows the formation of sugarcane fields with healthier plants, genetic standardization, and more homogeneous spatial arrangement [3]. These, therefore, increase crop yield when compared to the conventional system that uses whole stems for planting. Despite these advantages, sugarcane seedlings from the PSS system have low nutrient and water reserves for field transplantation and are very sensitive to water deficit after transplantation [4], causing seedling death and planting failures.

A strategy to reduce the effects of water deficit on PSS is the application of silicon (Si), as it has been used to mitigate different stresses in plants [5]. The role of Si in tolerance to water deficit is well known. It acts in physiological and biochemical processes such as maintenance of leaf water potential [6] and water content [7], preservation of photosynthetic pigments [8], adjustment of compatible osmolytes [9], and reduction of oxidative stress by decreasing cell electrolyte leakage index [10], thus benefiting growth parameters and dry biomass yield [11].

Silicon can also have an important structural role in plants under water deficit by changing the stoichiometric ratios of carbon (C), nitrogen (N) and phosphorus (P). Plant C: N: P stoichiometry has been widely investigated for ecological interactions, as these nutrients are strongly associated with plant biochemical and physiological functions [12]. Silicon changes C: N: P ratio because it is involved in ecosystem nutrient cycling, affecting absorption, distribution, and stoichiometry of nutrients such as N, P, and C [13, 14]. This is because Si needs less energy to be incorporated into leaf tissues than C does, and thus it can replace part of the C in cell-wall organic compounds, mainly for its high permeability in lipid bilayers [15]. Accordingly, Si improves C assimilation by plants since a large part of it can be incorporated into photosynthetically active tissues to the detriment of stabilizing compounds [14], whath can have C replaced with Si [15].

High Si-induced C metabolic efficiency can improve C skeletons production in plants and affect metabolism and nutritional efficiency of other structural nutrients such as N and P. These are also part of vital organic compounds for plants. However, Si-induced changes in C: N: P stoichiometry with increased nutritional efficiency have not yet been demonstrated in sugarcane PSS.

However, Si application method should be improved for use in seedling nurseries. For this purpose, foliar spraying is a practical technique because seedling nutrition is already carried out this way [4]. But as sugarcane belongs to the Poaceae family, which are considered as Si accumulators given the proteins in root tissues membranes that are specialized in its absorption and transport [16]. Therefore, Si is predominantly supplied via roots through nutrient solution [17], soil application [8], or substrate [13].

Foliar absorption occurs similarly among the different plant groups. Through, a process that initially requires penetration into the cuticle (passive percolation or surface adsorption) and then passes (active absorption) through the cells that make up the membranes (plasmalemma and tonoplast) [18]. Although foliar spraying has already been demonstrated in sorghum plants [19] and unstressed PSS [20], nothing is known about Si structural effect accumulated in leaf tissue after foliar spraying neither about C: N: P stoichiometry, which had already been evidenced for Si application to roots [13, 14, 21]. To our knowledge, no report has related PSS morphological and physiological parameters to changes in C: N: P stoichiometry for plants under water deficit. It is therefore interesting to verify this effect under different growing conditions and on plants with and without water deficit, as well as at different exposure times and stress levels.

Given the above, our study aimed to evaluate whether Si provided via root and foliar spraying changes C: N: P ratio and increases nutritional efficiency of PSS of sugarcane, besides evaluating whether water deficit interferes with such relationships.

## 2. Materials and methods

Three experiments were carried out in a greenhouse at the São Paulo State University (UNESP), Jaboticabal (Brazil), using pre-sprouted seedlings (PSS) of sugarcane. The first experiment was carried out without water stress and the other two experiments with short- and long-term water deficit. The experiments were analyzed independently in a randomized block design.

### 2.1 Experiment I

Treatments were carried out in a 2×3 factorial scheme and consisted of two sugarcane cultivars (CTC 4 [Sugarcane Technology Center] and RB 966928 [Inter-university Network for the Development of the Sugar-Energy Sector] and three Si supply forms (in nutrient solution via root [SiR], via foliar spraying [SiL], and one control with the absence of Si [-Si]), with seven replications.

The cultivars used were seleted because of their extensive use in growing areas and major agricultural traits such as high yield, improved health, ratoon regrowth ability, and adaptability to mechanized planting [22].

The experiment lasted the time recommended for PSS formation stage [4]. The experimental units consisted of 0.7-dm^3^ pots filled with washed sand. All pots had holes in the bottom and were placed on a plastic tray, where a nutrient solution was applied. The solution used was Hoagland and Arnon’s [23], with changes iniron concentration (368 μmol L^−1^) and source (Fe–EDDHMA), as indicated by Cavalcante et al. [24]. To avoid salt stress, the salt concentration in the nutrient solution was maintained at 25% of the stock solution during the first week of cultivation and then increased, to 50% from the second week onwards. The pH of the nutrient solution was maintained at 5.6±0.2 using 1.0 mol L^-1^ hydrochloric acid (HCl) or sodium hydroxide (NaOH) solutions.

The Si source was sodium and potassium silicate stabilized with sorbitol (113.4 g L^−1^ Si and 18.9 g L^−1^ K_2_O at a pH of 11.8). Silicon concentration was 2.0 mmol L^−1^ since in solution it starts to polymerize from 3.0 mmol L^-1^ [25]. This concentration was diluted in cultivation solution and supplied continuously during the experiment. Given the lack of a proper Si concentration for foliar application in a PSS, a preliminary test was carried out to test five concentrations (0, 1, 2, 3, and 4 mmol L^−1^), sprayed every four days for 34 days. The Si (x) concentrations has a quadratic effect on shoot dry matter yield (y) (y = −0.45x^2^** + 3.063x + 7.65; R^2^ = 0.97), with a maximum point at 3.4 mmol L^−1^. Therefore, this was the concentration used for foliar spraying in experiments.

In short, five Si foliar sprays were done every four days (at 12, 16, 20, 24, and 28 days after emergence—DAE). The spray solution pH was adjusted to 5.5±0.2, and the K content in Si source was balanced using a 1.0 mol L^−1^ potassium chloride (KCl) solution.

The seedlings were cut at 36 DAE when they had six fully developed leaves. The cutting was made at 30 cm from the base of the last fully developed leaf, which represented about one-third of the leaves. Morever, two-thirds of the shoot were collected for leaf chemical analysis.

### 2.2 Experiment II

Treatments were conducted in a 3×2 factorial scheme and consisted of the same supply forms used previously (SiR, SiL and -Si), associated with water deficit (−0.6 MPa [water deficit—WD]) and its without water deficit (control), with six replications. In this assay, seedlings of the cultivar RB 966928 were used.

This experiment was conducted in two steps. In the first, Si was applied to seedlings at the formation stage as in Experiment I (item 2.1). Then, the seedlings were transplanted to polypropylene pots with 8-dm^3^ Hoagland and Arnon’s nutrient solution [23], which was oxygenated (air bubbler). The plants were grown under these conditions for ten days for adaptation after transplantation.

In the second step, WD was applied using polyethylene glycol (PEG-6000), which is not toxic to plants and induce WD by decreasing solution osmotic potential [26]. This procedure enables measuring plant responses within the first hours of water restriction. The concentration of PEG-6000 used was enough to induce an osmotic potential of -0.6 MPa [27] in cultivation solution, which is considered strong for Poaceae family plant [6]. The control (no water deficit) contained only deionized water. After three days under water stress and lack of Si supply, the plants were collected for leaf tissue analysis.

### 2.3 Experiment III

Treatments were arranged in a 3×3 factorial scheme and consisted of the same Si supply forms (SiR, SiL and -Si) associated with three soil water availability levels (70% [control], 50% [moderate WD], and 30% [severe WD] of soil water retention capacity–WRC), with six replications. In this assay, seedlings of the cultivar CTC 4 were used.

This experiment was also conducted in two steps. The first consisted of applying Si to seedlings both in SiR and SiL, as in Experiment I (item 2.1). Then, the seedlings were transplanted to 7-dm^3^ pots filled with 5.5 dm^3^ dystrophic Oxisol from *Ap* horizon [28]. In the second, WD was applied to the seedlings.

Soil chemical analysis was carried out for fertility purposes as in Raij et al. [29]. The results were as follow: pH CaCl_2_ = 5, organic matter = 11 g dm^−3^, P (resin) = 11 mg dm^−3^, S = 18 mg dm^−3^, Ca = 11 mmol_c_ dm^−3^, Mg = 6 mmol_c_ dm^−3^, K = 1.9 mmol_c_ dm^−3^; Al = 0 mmol_c_ dm^−3^; H+Al = 20 mmol_c_ dm^−3^; sum of bases = 18.5 mmol_c_ dm^−3^; cation exchange capacity = 38.1 mmol_c_ dm^−3^; and aluminum saturation = 0%. Silicon content (3.0 mg dm^−3^) was determined as in Korndörfer et al. [30]. Soil particle size distribution consisted of 540, 380, and 90 g kg^−1^ of sand, clay, and silt, respectively.

Limestone with a total neutralizing valueof 125% (CaO of 48%, and MgO of 16%; 0.285 g dm^−3^) was applied and incorporated into the soil to raise its base saturation (V%) to 60%. Thirty days after liming, the soil was fertilized by incorporating 150 mg dm^−3^ N, P, and K as ammonium sulfate, triple superphosphate, and potassium chloride, respectively, and 5 mg dm^−3^ Zn as zinc sulfate.

Soil water availability was set as its microporosity obtained by the tension table method, with a 60-cm water column. To this end, undisturbed soil samples were collected using a volumetric ring (98.125 cm^3^), saturated for 24 h, placed on a tension table for 72 h, and weighed for mass determination (a). Afterwards, the samples were oven-dried at 110°C for 24 h and reweighed (b) [31].

Total microporosity (Mi = [a–b]/V) was 0.3036 cm^3^ cm^−3^, which is equivalent to 100% of the soil WRC. However, the ideal water condition (control) for sugarcanewas 70% of that value (Mi = 0.2125 cm^3^ cm^−3^), as it allows 70% of the micropores to be filled with available water and the remaining 30% with air, maintaining gas exchange in the roots zone [32]. Since severe WD can be reached at 30% soil WRC, as in previous studieswith sugarcane [32], we set as moderate WD aticit 50% soil WRC, as the mean between control (70%) and severe WD (30%).

Water availability was controlled daily by weighing the pots and replacing losses. In this experiment, WD lasted 30 days for being the most critical period after transplanting seedlings to the field.

### 2.4 Analyses

#### 2.4.1 Relative water content

Leaf relative water content in plants under WD was determined using three leaf discs (~ 129 mm^2^) from the first fully developed leaves. It was estimaded by the equation proposed by Barrs and Weatherley [33].

#### 2.4.2 Electrolyte leakage index

Electrolyte leakage index was calculated using the formula proposed by Dionisio-Sese and Tobita [34]. It was determined using three discs (~129 mm^2^) from the first fully developed leaves. This index was used to estimate cell-membrane integrity damages in plants under WD.

#### 2.4.3 Chlorophyll quantification

Content of chlorophyll a and b (Chl a+b) was determined in 6-mg leaf discs from the middle third of the first fully-developed leaf. These were collected from plants grown under WD, and determinations followed the method proposed by Lichtenthaler [35].

#### 2.4.4 Plant height

Plant height was measured from the plant base to the tip of the last fully expanded leaf, in plants under WD.

#### 2.4.5 Dry matter yield

Plants from the three experiments were sampled and washed in running water, detergent solution (0.1% v/v), HCl solution (0.3% v/v), and deionized water. The plant material was dried in a forced-air circulation oven (65±5°C) until constant mass and then weighed on an analytical scale for dry matter yield measurement.

#### 2.4.6 Silicon concentration

Total Si concentration in plant shoot was determined by extracting it using the method described by Kraska and Breitenbeck [36] and reading by the colorimetric method using a spectrophotometer at 410 nm, as in Korndörfer et al. [30].

#### 2.4.7 Carbon, nitrogen, and phosphorus concentrations

Total C and N concentrations in plant shoot were determined by dry combustion at 1000°C using a LECO Truspec CHNS elemental analyzer, calibrated with a LECO 502–278 wheat standard (C = 45% and N = 2.68%). Total P concentration was determined using the colorimetric molybdenum antimony method in a spectrophotometer, as in Bataglia et al. [37].

#### 2.4.8 Efficiency of C, N, and P uses

Accumulations of C, N, and P in plant shoot were estimated based on their concentrations and plant dry matter. Afterwards, C, N and P use efficiencies (CUE, NUE and PUE, respectively) were calculated according to Fageria and Baligar [38].

### 2.5 Statistical analysis

After variance homogeneity by the Shapiro-Wilk W-test, all data were submitted to bidirectional analysis of variance (ANOVA) by the F-test (p≤0.05). The experiments were analyzed separately. Averages were compared by the Tukey’s test (p≤0.05), using the statistical software SAS^®^ (Cary, NC, USA).

The data were also subjected to hierarchical cluster analysis. For this purpose, data were standardized by the following equation: Z_ij_ = (X_ij_-X_j_)/S_j_, wherein _j_ = number of variables; _i_ = number of treatments; Z_ij_ = standardized value of X_ij_; X_j_ and S_j_ = mean and standard deviation of the variables, respectively. Euclidean distance was used as a similarity coefficient and the UPGMA method (unweighted pair-group method using arithmetic averages) as a group connection algorithm. The statistical tests were performed using the free software environment R and the package “pheatmap”.

## 3. Results

### 3.1 Plant Si concentration

**Fig 1:**
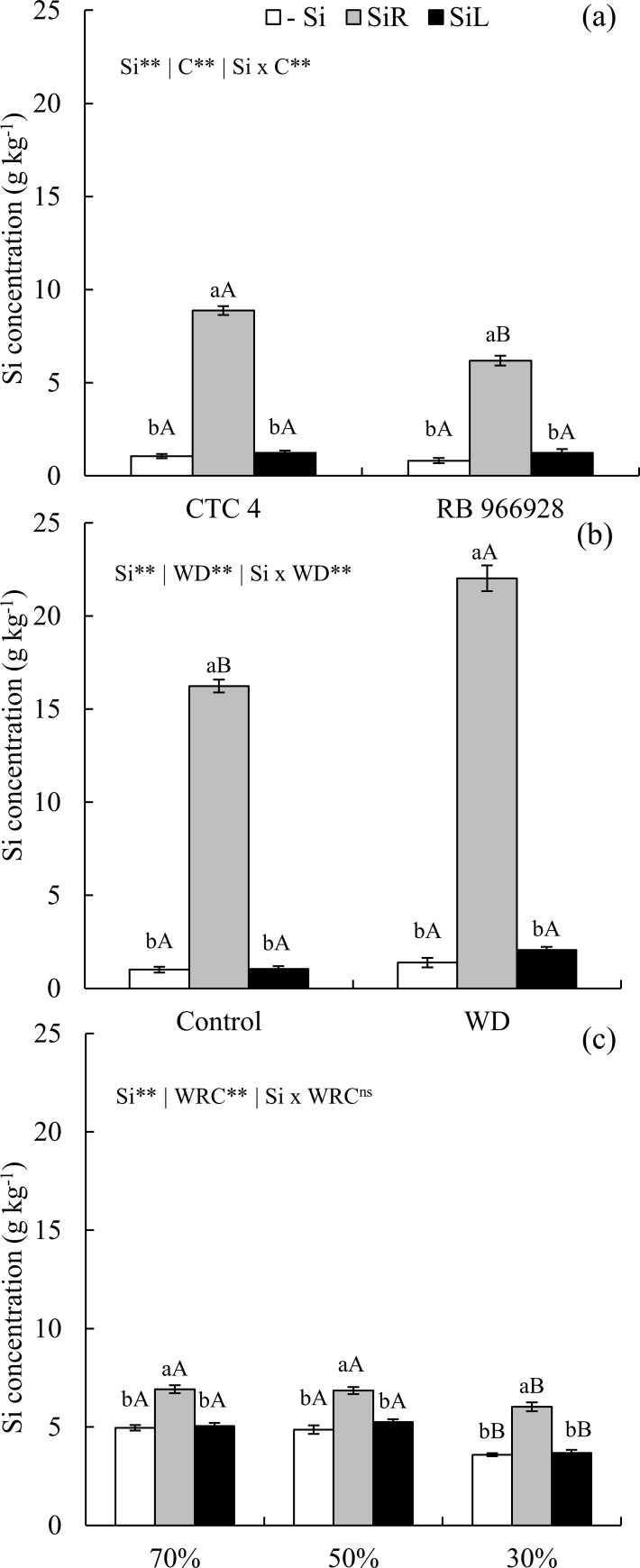
Concentration of silicon (Si) supplied via nutrient solution (SiR), foliar spraying (SiL), and in the absence (−Si) in two pre-sprouted sugarcane seedling cultivars (CTC 4 and RB 966928) without water deficit (a); under short-term water deficit (WD) induced by polyethylene glycol (−0.6 MPa) plus the control (absence of water deficit) (b), and under long-term water deficit induced by levels of soil water retention capacity (WRC) (70, 50, and 30%) (c). ** and *: significant at 1 and 5% probability, respectively; ^ns^: non-significant by the F-test. Lowercase letters show differences in Si forms and uppercase letters between cultivars (a), WD (b) and WRC (c), by the Tukey’s test. Bars represent the standard error of the mean.

When SiR was used, Si concentration increased in PSS shoot under no water stress, mainly for the cultivar CTC 4 (Fig 1A). Seedlings under short-term WD showed higher Si concentrations in SiR, with a marked increase in plants under WD (Fig 1B). Morever, Si concentration increased in SiR for plants under long-term WD for all levels of soil water retention capacity (Fig 1C).

### 3.2 Concentrations; stoichiometric relationships; C, N, and P use efficiencies; and dry matter yield–Experiment I

**Fig 2:**
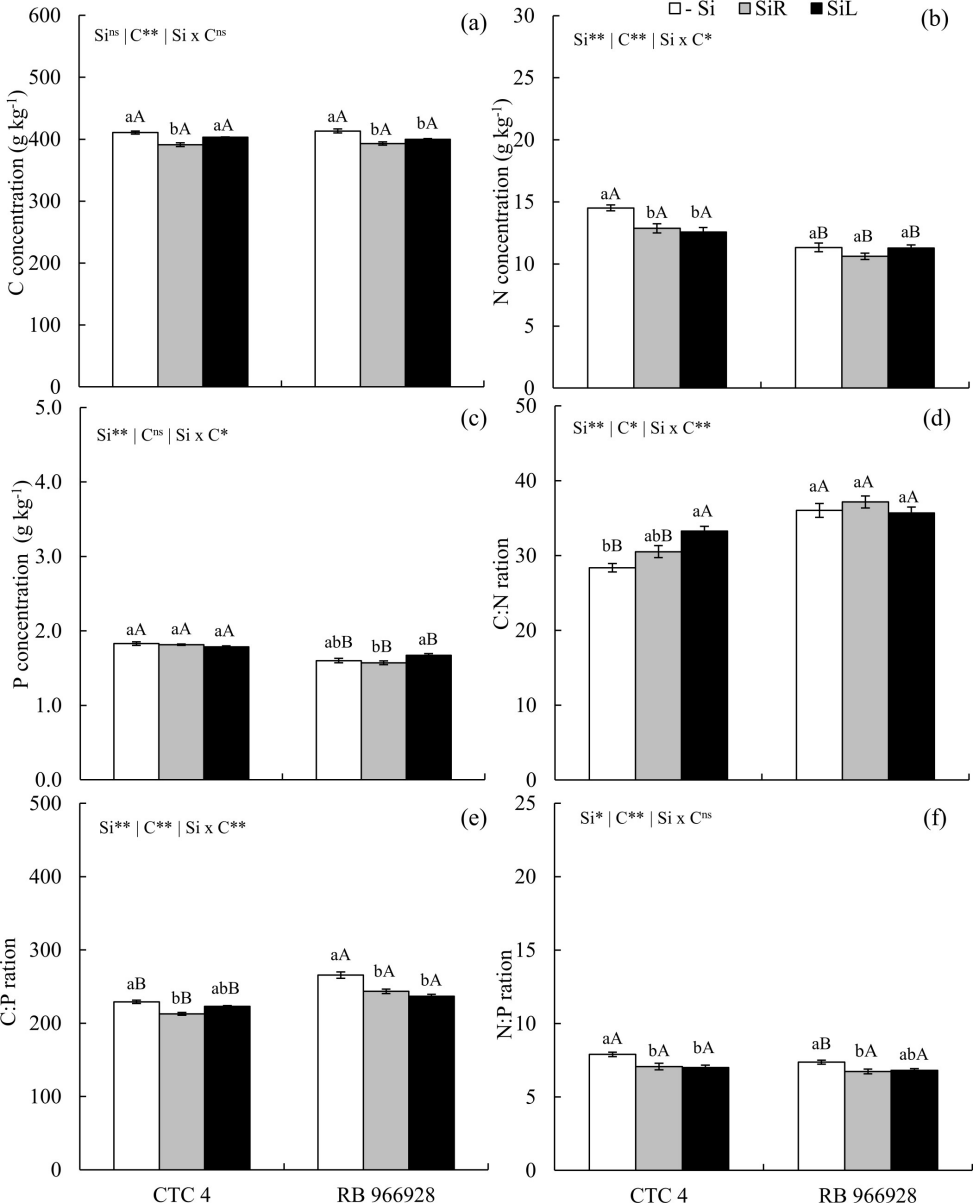
Concentrations of C (a), N (b), and P (c), ratios C: N (d), C: P (e) and N: P (f) in two pre-sprouted sugarcane seedling cultivars (CTC 4 and RB 966928) under three forms of Si supply (Si via nutrient solution [SiR], Si via foliar spraying [SiL], and Si absence [-Si]). ** and *: significant at 1 and 5% probability, respectively; ^ns^: non-significant by the F-test. Lowercase letters show differences in Si forms and uppercase letters between cultivars by the Tukey’s test. Bars represent the standard error of the mean; n = 7.

Carbon concentration decreased with Si supply in SiR in both cultivars and SiL in RB 966928 (Fig 2A). Nitrogen concentration decreased in CTC 4 with Si supplied via both forms (Fig 2B). Phosphorus concentration was higher with Si supply in SiL in cultivar RB 966928 compared to the supply in SiR (Fig 2C). Foliar spraying of Si increased the C: N ratio in CTC 4 (Fig 2D); hovewer, the C: P ratio decreased in both supplied forms for RB 966928 and only in SiR for CTC 4 (Fig 2E). The relationship between N and P also decreased in both Si supplied forms in CTC 4 and only in SiR for RB 966928 (Fig 2F).

**Fig 3:**
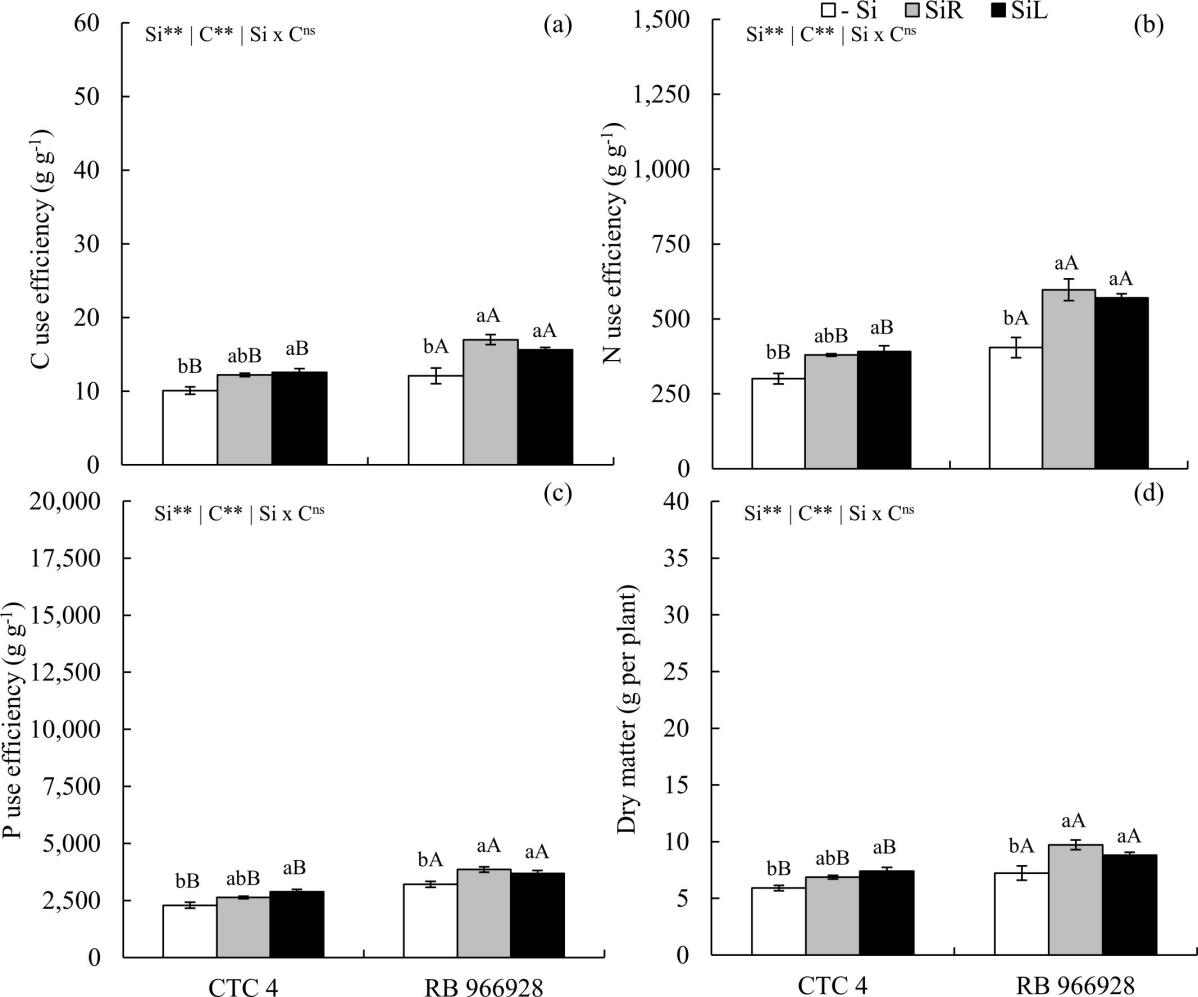
Use efficiencies of C (a), N (b), P (c) and dry matter yield (d) in two sugarcane pre-sprouted seedling cultivars (CTC 4 and RB 966928) under three forms of Si supply (Si via nutrient solution [SiR], Si via foliar spraying [SiL], and Si absence [-Si]). ** and *: significant at 1 and 5% probability, respectively; ^ns^: non-significant by the F-test. Lowercase letters show differences in Si forms and uppercase letters between cultivars by the Tukey’s test. Bars represent the standard error of the mean; n = 7.

CUE increased with Si supply in SiR and SiL by up to 21 and 20% in CTC 4 and 41 and 29% in RB 966928, respectively (Fig 3A). NUE and P PUE increased with Si supply in SiL in CTC 4 and via both supply forms in RB 966928 (Fig 3B and 3C). Dry matter yield had a similar effect as in nutrient use efficiency, with increases of 16 and 25% in CTC 4 and 34 and 22% in RB 966928, for Si supply in SiR and SiL, respectively (Fig 3D).

**Fig 4:**
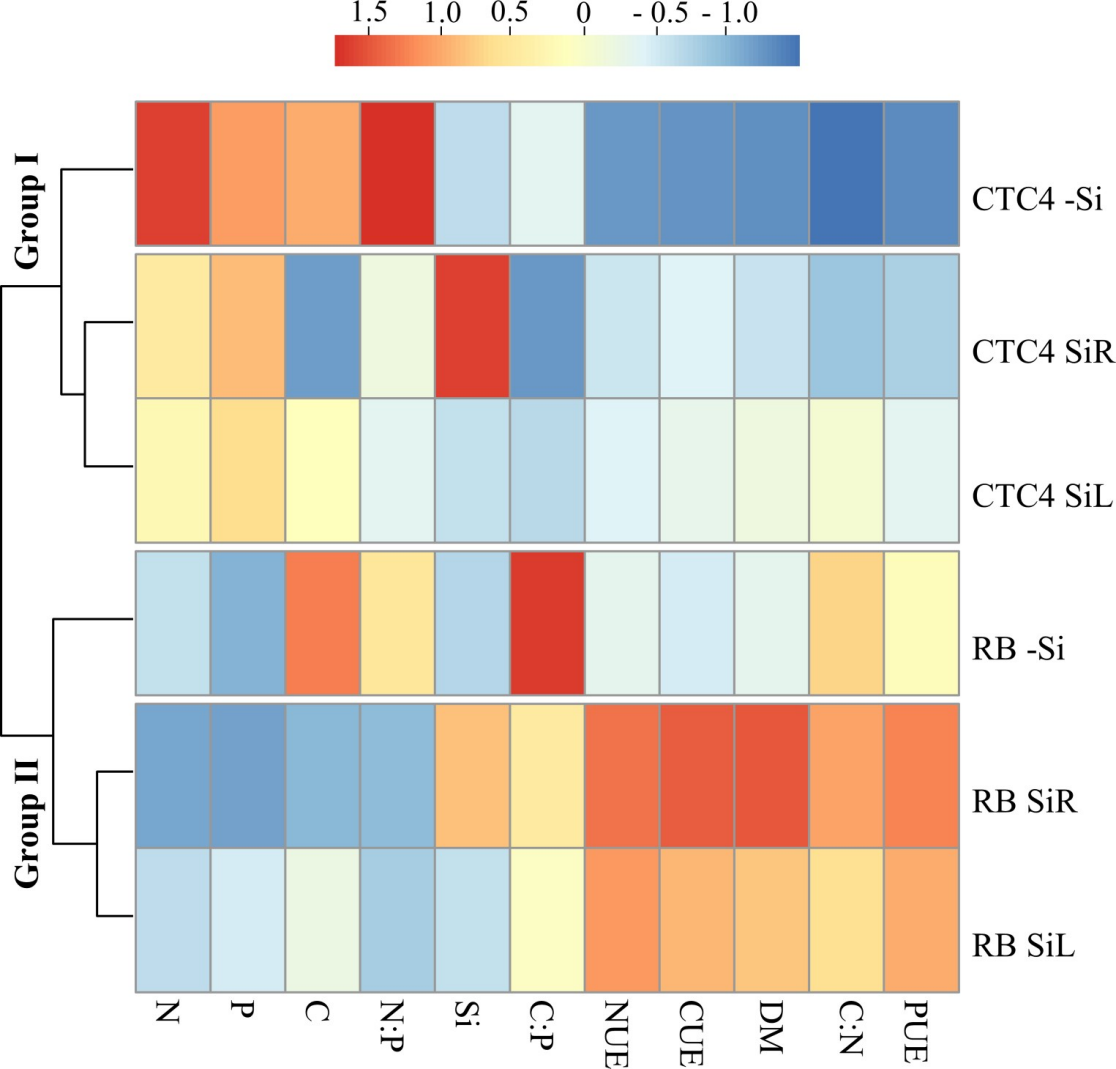
Hierarchical cluster analysis with standardized data of Si, C, N, and P concentrations; C: N, C: P, and N: P ratios; use efficiencies of C (CUE), N (NUE), P (PUE) and dry matter production (DM) for two sugarcane pre-sprouted seedling cultivars (CTC 4 and RB 966928) under three forms of Si supply (Si via nutrient solution [SiR], Si via foliar spraying [SiL], and Si absence [-Si]).

The cluster analysis showed that cultivars were classified into different groups. For both cultivars, Si-supplied plants both form SiR and SiL were grouped but separated from those in -Si. After data standardization, CTC 4 seedlings under -Si had higher N and C concentrations than those supplied both SiR and SiL. Yet for RB 966928 seedlings, Si supply effect was only evident for C concentration, with higher amount in -Si plants. However, for both cultivars, although Si concentration increased only in SiR, its increasing effects on CUE, NUE, PUE and dry matter yield were very similar between both application forms if compared to -Si (Fig 4).

### 3.3 Concentrations; stoichiometric relationships; morphological and physiological parameters; C, N, and P use efficiencies; and dry matter yield–Experiment II

**Fig 5:**
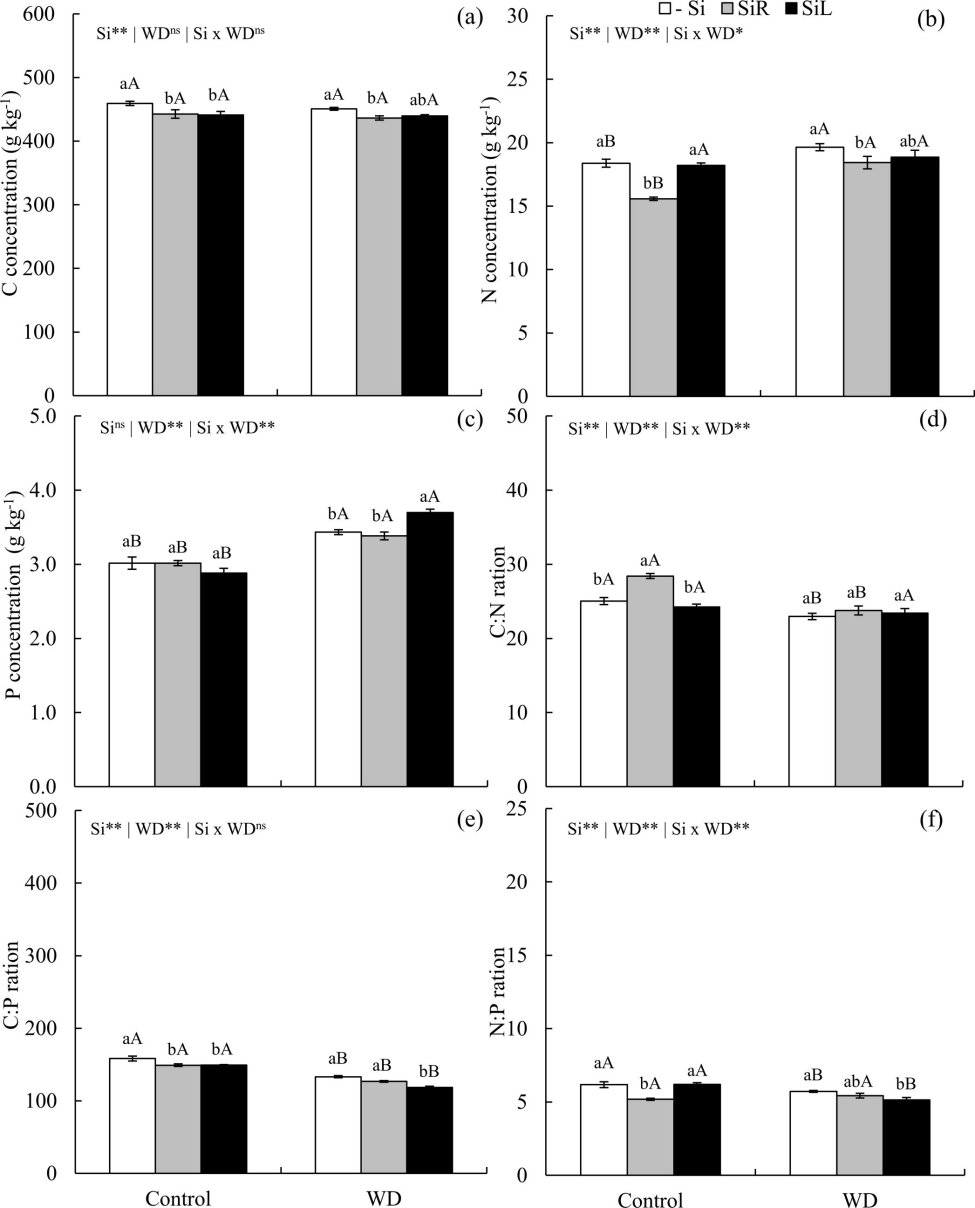
Concentrations of C (a), N (b), and P (c), ratios C:N (d), C:P (e) and N:P (f) in pre-sprouted sugarcane seedlings under absence (control) and presence (−0.6 MPa) of water deficit (WD) short-term and under three forms of Si supply (Si via nutrient solution [SiR], Si via foliar spraying [SiL], and Si absence [-Si]). ** and *: significant at 1 and 5% probability, respectively; ^ns^: non-significant by the F-test. Lowercase letters show differences in Si forms and uppercase letters between WD levels by the Tukey’s test. Bars represent the standard error of the mean; n = 6.

Carbon concentration decreased in plants under without water deficit with Si supply via both forms and in plants under short-term WD with Si supplied in SiR (Fig 5A). Nitrogen decreased in plants under both water conditions with Si supplied in SiR (Fig 5B). WD increased P concentration in plant shoot, and Si supply in SiL intensified increase (Fig 5C). The C: N ratio increased in plants supplied with Si in SiR under suitable water condition (Fig 5D). Yet the C: P ratio decreased in unstressed plants with Si supplied via both forms, as well as in plants under WD with Si supplied in SiL (Fig 5E). The N: P ratio decreased in SiR unstressed plants and SiL stressed plants (Fig 5F).

**Fig 6:**
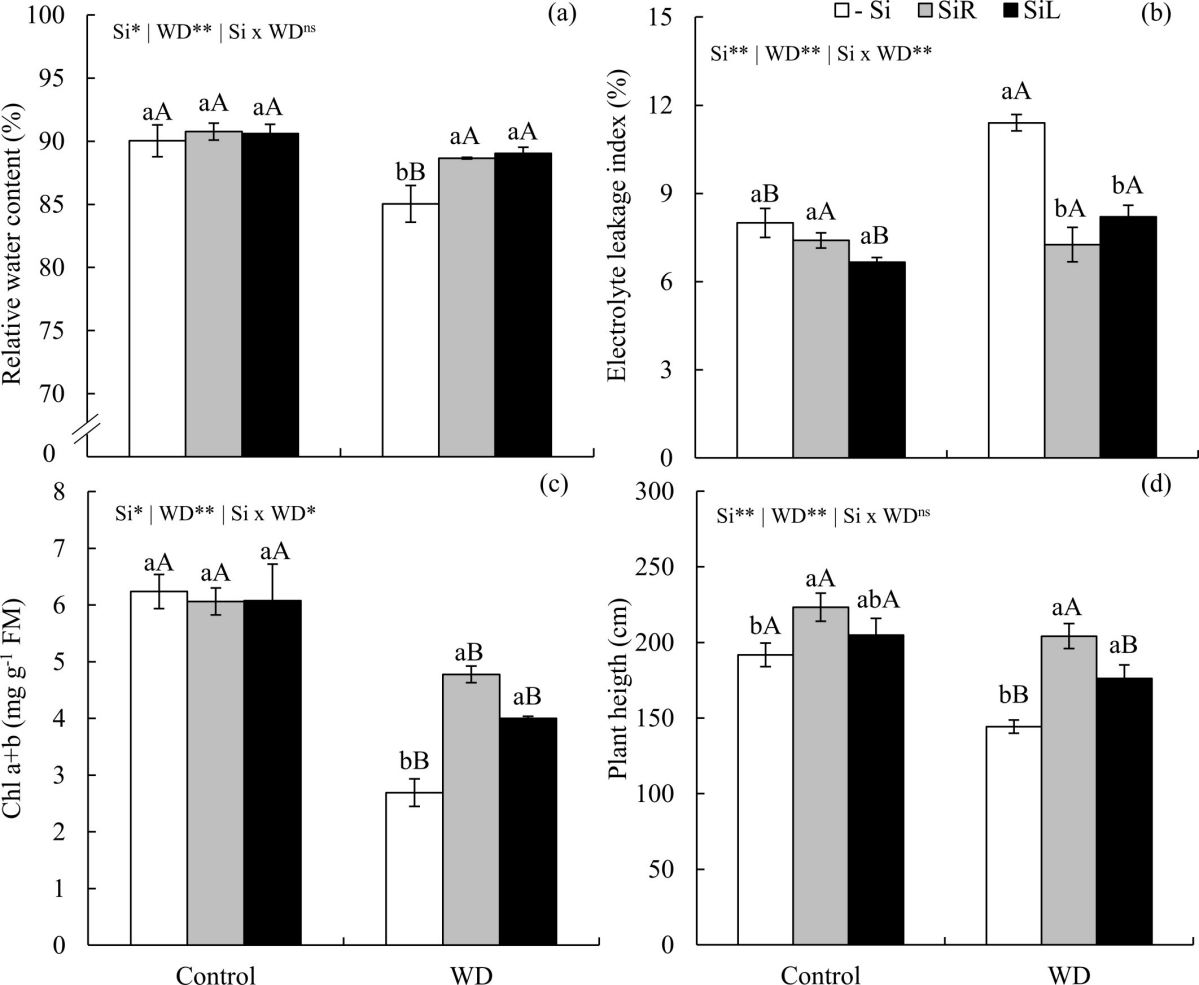
Relative water content (a), electrolyte leakage index (b), chlorophyll (Chl) a+b (c), plant height (d) of pre-sprouted sugarcane seedlings under absence (control) and presence (−0.6 MPa) of water deficit (WD) in the short-term and under three forms of Si supply (Si via nutrient solution [SiR], Si via foliar spraying [SiL], and Si absence [-Si]). ** and *: significant at 1 and 5% probability, respectively; ^ns^: non-significant by the F-test. Lowercase letters show differences in Si forms and uppercase letters between WD levels by the Tukey’s test. Bars represent the standard error of the mean; n = 6.

Short-term water stress decreased water content in leaf tissue of plants in -Si. Both SiR and SiL increased leaf water content in plants under WD as in unstressed plants (Fig 6A). Electrolyte leakage index increased of plants in -Si under WD. In turn, both forms of Si supply maintained lower leakage indexes in plants under short-term WD (Fig 6B). Chl a + b content decreased by 57% in plants under WD compared to the control, but increased when Si was supplied via both forms (Fig 6C). In plants in -Si short-term WD decreases height. However, Si supply via both forms promoted taller plants when under short-term WD (Fig 6D).

**Fig 7:**
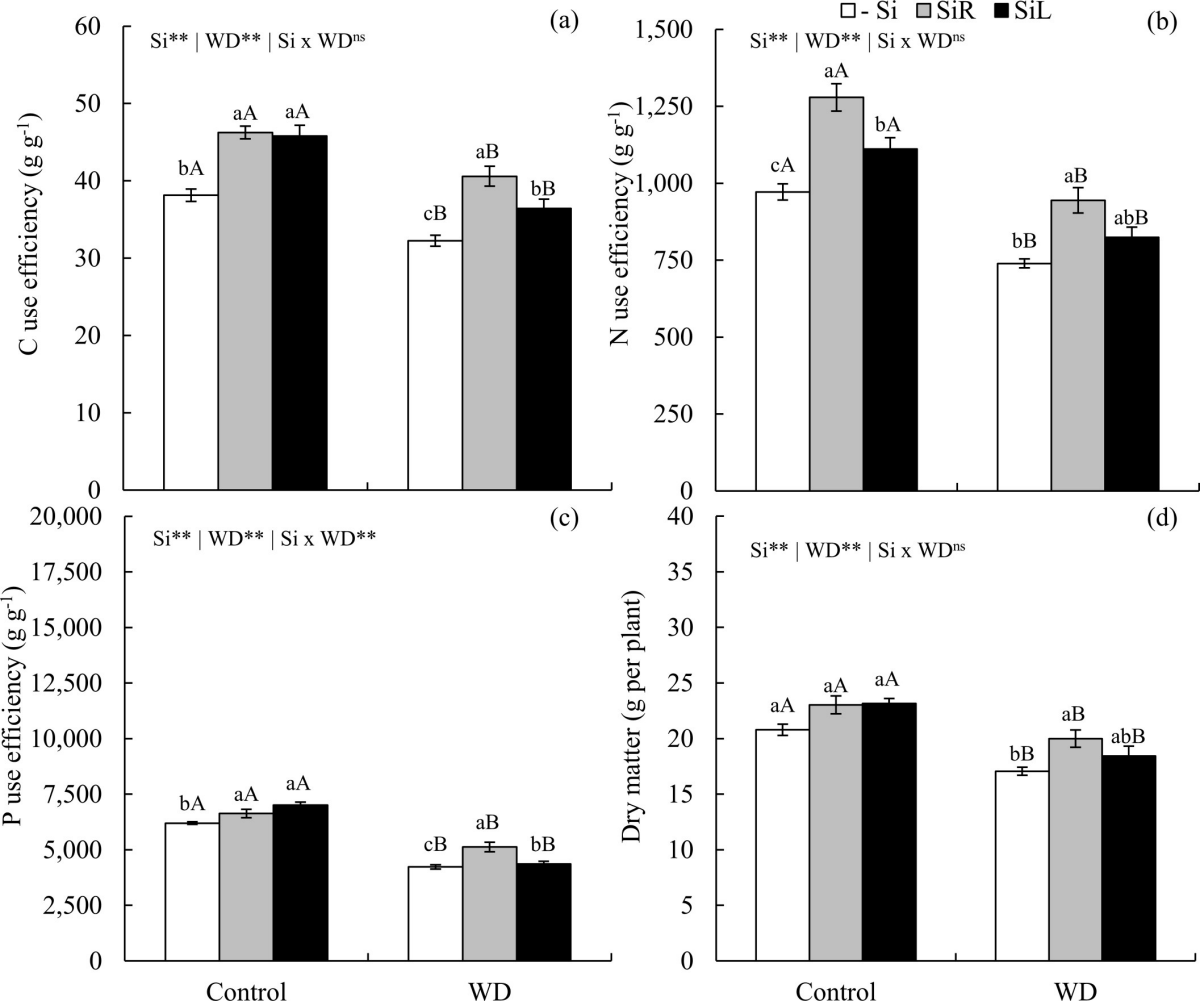
Use efficiencies of C (a), N (b), and P (c) and dry matter yield (d) in pre-sprouted sugarcane seedlings under absence (control) and presence (−0.6 MPa) of water deficit (WD) in the short-term and under three forms of Si supply (Si via nutrient solution [SiR], Si via foliar spraying [SiL], and Si absence [−Si]). ** and *: significant at 1 and 5% probability, respectively; ^ns^: non-significant by the F-test. Lowercase letters show differences in Si forms and uppercase letters between WD levels by the Tukey’s test. Bars represent the standard error of the mean; n = 6.

Short-term WD decreased CUE in plants -Si. But when Si was provided, it increased by 26 and 13% in SiR and SiL, respectively. In unstressed plants, Si also improved CUE, increasing by up to 21 and 20%, with Si input in SiR and SiL, respectively, compared to -Si (Fig 7A). Besides, NUE also decreased under WD in plants -Si, but Si supply increased it both in plants with or without WD, mainly when supplied in SiR (Fig 7B). Both forms of Si supply also increased the relation between dry matter gain and P accumulation in the control treatment and stressed plants (Fig 7C). Plant biomass under WD decreased and Si suplly increased it up to 17% in SiR and by up to 8.0% in SiL, in relation to -Si (Fig 7D).

**Fig 8:**
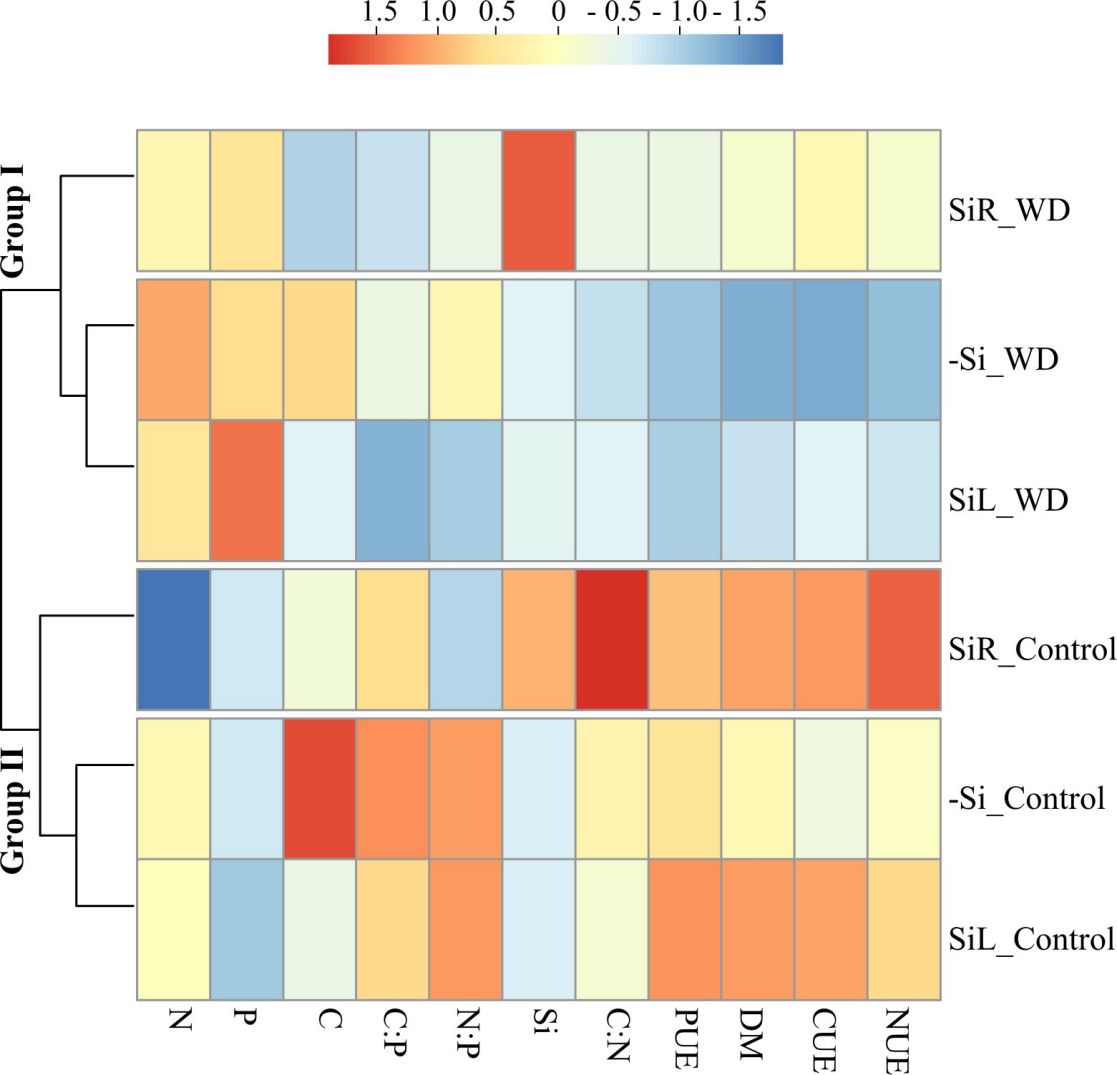
Hierarchical cluster analysis with standardized data of Si, C, N, and P concentrations, C: N, C: P, and N: P ratios; use efficiencies of C (CUE), N (NUE), P (PUE) and dry matter yield (DM) for pre-sprouted sugarcane seedlings under absence (control) and presence (−0.6 MPa) of water deficit (WD) in the short-term and under three forms of Si supply (Si via nutrient solution [SiR], Si via foliar spraying [SiL], and Si absence [−Si]).

Cluster analysis divided seedlings with or without WD into groups. Under both conditions, seedling supplied via SiR were classified in a distinct group from -Si and SiL. Standardized data analysis showed that Si supply via SiR decreased N concentrations in plants under both water conditions. Although Si concentration increased only with SiR supply, SiL decreased C concentration and increased CUE, NUE and PUE and dry matter yield, regardless of the water stress. Therefore, Si was highly efficient in providing structural changes even at low Si accumulations (Fig 8).

### 3.4 Concentrations; stoichiometric relationships; morphological and physiological parameters; C, N, and P use efficiencies; and dry matter yield–Experiment III

**Fig 9:**
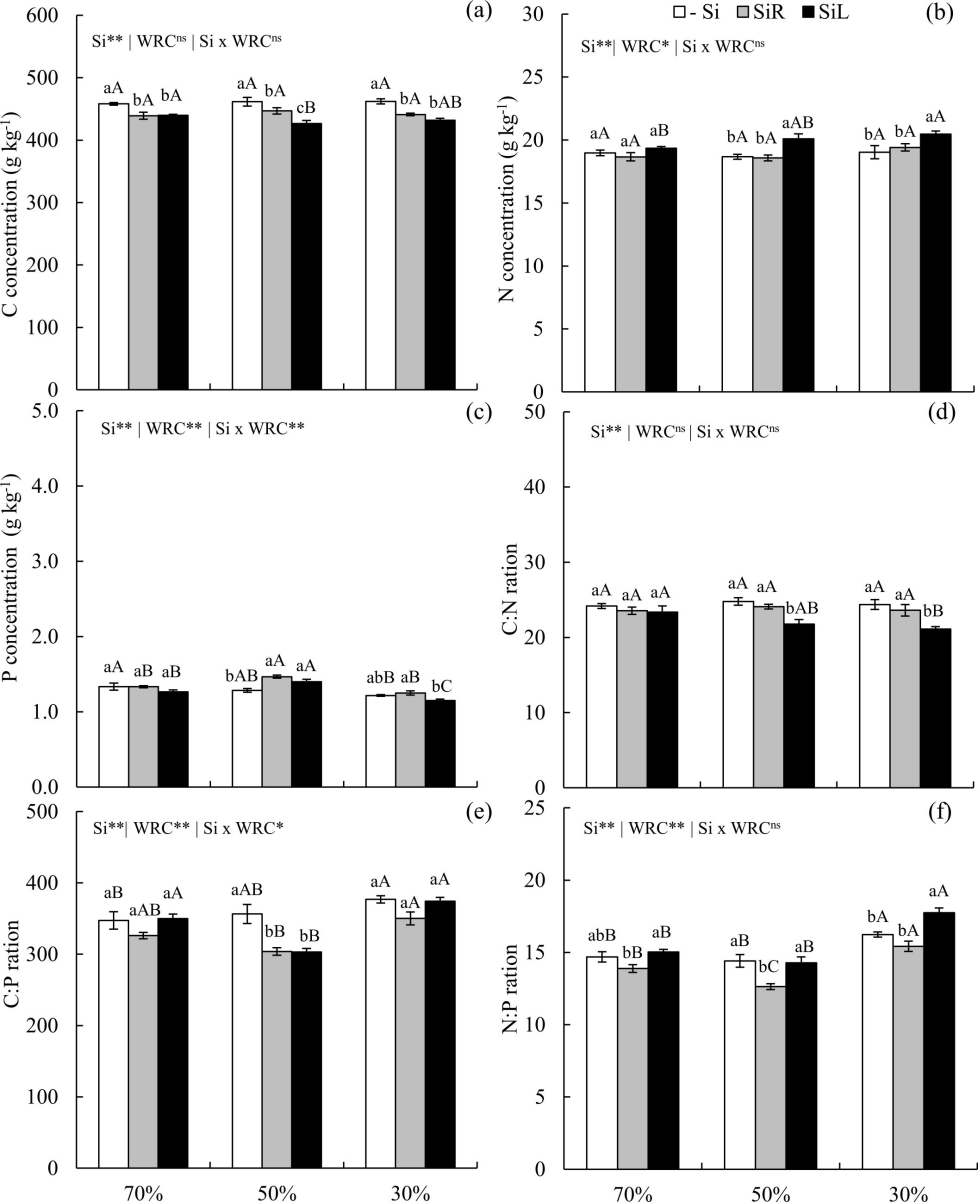
Concentrations of C (a), N (b), and P (c), ratios C: N (d), C: P (e) and N: P (f) in pre-sprouted sugarcane seedlings under three soil water retention capacity (WRC) levels (70, 50, and 30%) and three forms of Si supply (Si via nutrient solution [SiR], Si via foliar spraying [SiL], and Si absence [−Si]). ** and *: significant at 1 and 5% probability, respectively; ^ns^: non-significant by the F-test. Lowercase letters show differences in Si forms and uppercase letters in WRC levels by the Tukey’s test. Bars represent the standard error of the mean; n = 6.

Carbon concentration decreased in plants supplied with Si via SiR and SiL under all water conditions (Fig 9A). SiL increased N under 50 and 30% WRC of the soil, with higher levels in plants under more severe water restriction (Fig 9B). Phosphorus concentration increased in plants under both Si supply at 50% WRC of the soil (Fig 9C). SiL decreased the C: N ratio at 50 and 30% WRC of the soil (Fig 9D). Yet the C: P ratio decreased in both forms of Si supply at only 50% WRC of the soil (Fig 9E). Moreover, the N: P ratio decreased with Si supply in via SiR at all WRC levels and increased with Si supply in via SiL under severe WD (Fig 9F).

**Fig 10:**
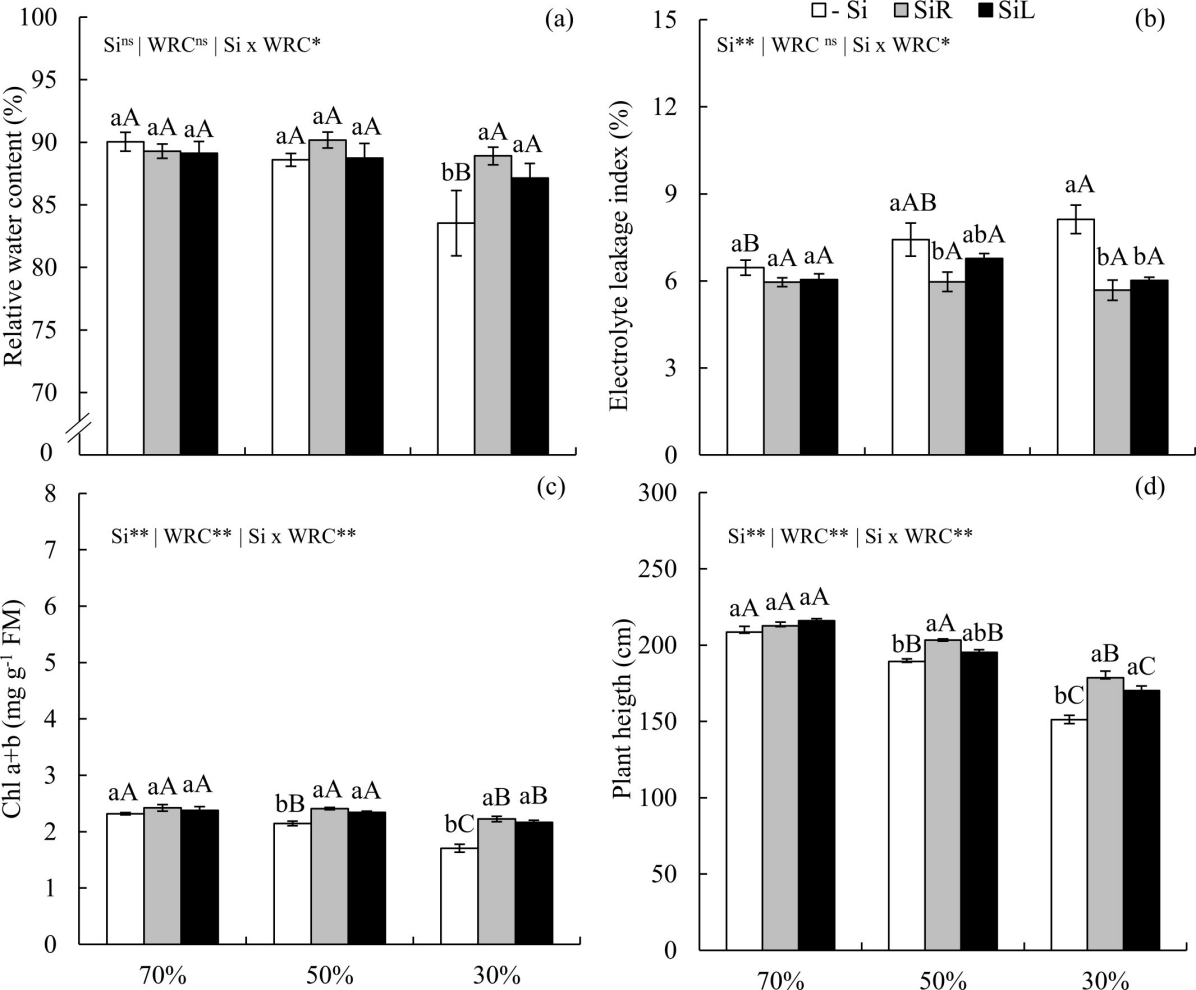
Relative water content (a), electrolyte leakage index (b), chlorophyll (Chl) a+b (c), plant height (d) of pre-sprouted sugarcane seedlings under three soil water retention capacity (WRC) levels (70, 50, and 30%) and three forms of Si supply (Si via nutrient solution [SiR], Si via foliar spraying [SiL], and Si absence [−Si]). ** and *: significant at 1 and 5% probability, respectively; ^ns^: non-significant by the F-test. Lowercase letters show differences in Si forms and uppercase letters in WD levels by the Tukey’s test. Bars represent the standard error of the mean; n = 6.

Leaf water content decreased in -Si plants under severe WD when compared to the suitable water condition. Silicon suplly either via SiR or SiL increased water content in plants under severe WD, equaling those unstressed plants (Fig 10A). Electrolyte leakage index increased with soil water stress levels in -Si plants. SiR promoted lower indexes when compared to the −Si under moderate and severe WDs. However, SiL had the same effect only under severe WD (Fig 10B).

In -Si, Chl a+b decreased with increasing water restriction levels. Both Si supply forms increased Chl a+b contents in plants under moderate and severe WDs (Fig 10C). In -Si, soil water content reductions decreased plant heights. Silicon increased plant height via SiR under moderate and severe WD and via SiL under severe WD (Fig 10D).

**Fig 11:**
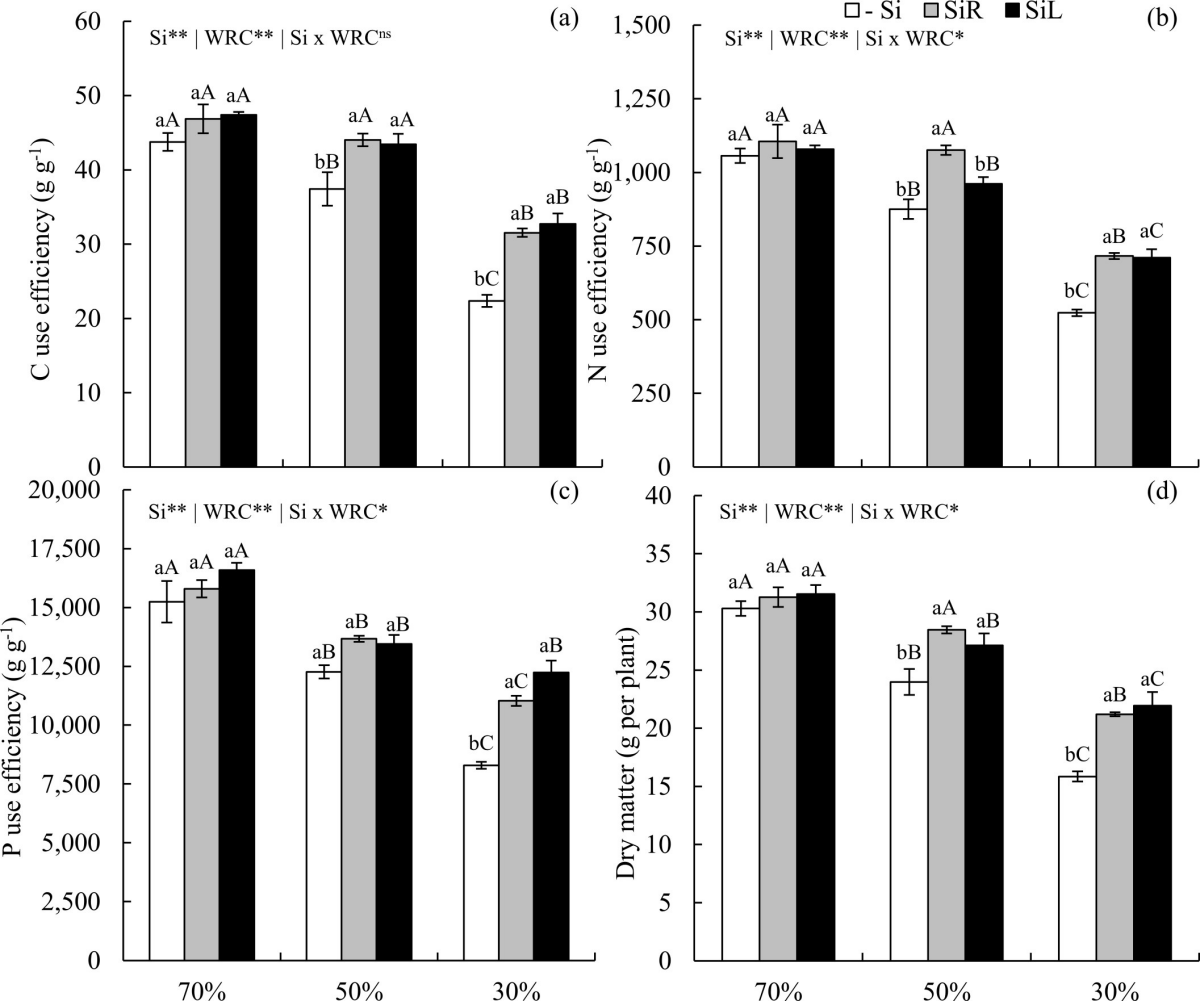
Use efficiencies of C (a), N (b), and P (c) and dry matter yield (d) in pre-sprouted sugarcane seedlings under three soil water retention capacity (WRC) levels (70, 50, and 30%) and under three forms of Si supply (Si via nutrient solution [SiR], Si via foliar spraying [SiL], and Si absence [−Si]). ** and *: significant at 1 and 5% probability, respectively; ^ns^: non-significant by the F-test. Lowercase letters show differences in Si forms and uppercase letters in WRC levels by the Tukey’s test. Bars represent the standard error of the mean; n = 6.

CUE decreased in -Si plants under WD condition. However, Si supply increased it by up to 18 and 41% via SiR and 16 and 46% via SiL at 50 and 30% WRC of the soil, respectively, compared to -Si at same WRC level (Fig 11A). NUE also had an increase with Si supply via SiR in plants under moderate WD and via both forms in those under severe WD (Fig 11B). Moreover, both Si supply forms similarly increased PUE only in plants under severe WD when compared to the −Si (Fig 11C).

Biomass production was strongly affected by an increase in Si concentration, demonstrating effects on CUE, NUE and PUE, with increases of 19 and 34% with Si supply via SiR and 13 and 38% via SiL, at 50 and 30% WRC of the soil, respectively, compared to -Si at same WRC level (Fig 11D).

**Fig 12:**
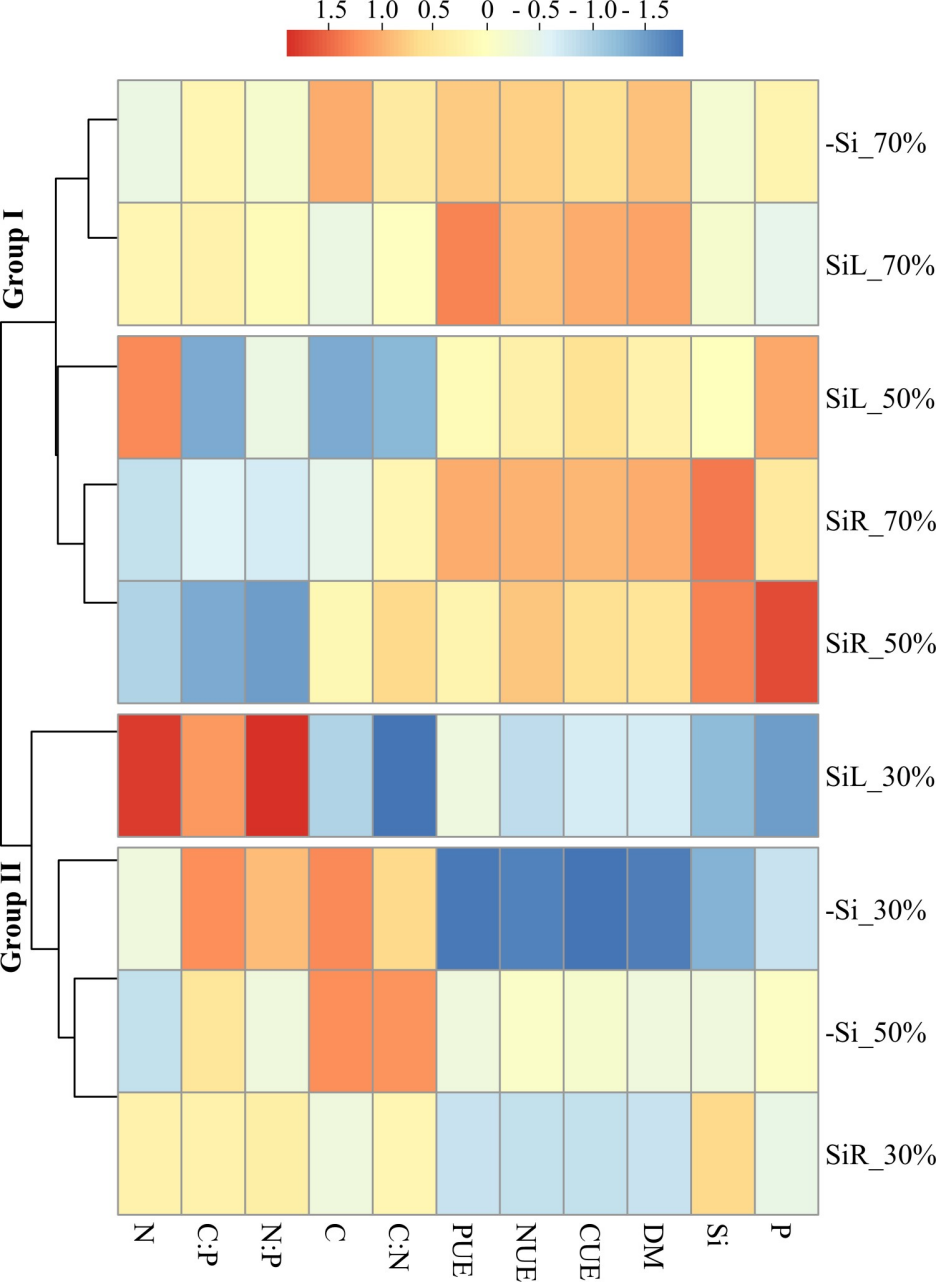
Hierarchical cluster analysis with standardized data of Si, C, N, and P concentrations; C:N, C:P, and N:P ratios; use efficiencies of C (CUE), N (NUE), P (PUE); and dry matter yield (DM) for pre-sprouted sugarcane seedlings under three soil water retention capacity (WRC) levels (70, 50, and 30%) and under three forms of Si supply (Si via nutrient solution [SiR], Si via foliar spraying [SiL], and Si absence [−Si]).

The cluster analysis divided the plants into groups according to sensitivity to damage under different levels of WD. One group consisted of plants under control condition (70% WRC) and plants receiving Si via SiR or via SiL under moderate WD (50% WRC). Another group composed of plants under severe WD (30% WRC) and plants under moderate WD but -Si. Standardized data analysis showed that plants in -Si had higher C concentration at all WD levels. Even though SiR increased Si concentration, SiL also had beneficial effects, especially increasing CUE, NUE and PUE, reflected in dry matter yield (Fig 12).

## 4. Discussion

Silicon supply improved the C: N: P stoichiometric ratio, CUE, NUE and PUE and dry matter accumulation of PSS of sugarcane. Such effects is influenced by application method and water deficit condition. The effect can be obtained either by supply in SiR or SiL, even in plants with active Si absorption such as sugarcane.

Sugarcane belongs to the family Poaceae and is classified as Si accumulator since it has proteins specialized in Si active absorption and transport in roots [16]. For this reason, Si accumulation was high with supply in SiR (Fig 1), changing C, N and P concentrations and their ratios (Figs 2, 5 and 9), as well as increasing their use efficiencies and hence dry matter yield (Figs 3, 7 and 11). Changes in C: N: P stoichiometric relationships with Si supply via roots have already been observed in plants of *Phragmites australis* [13], wheat [12] and even sugarcane [21], without WD. However, our results highlight that Si supply also raises the use efficiency of these nutrients and that it occurs in plants under WD.

Silicon accumulation by SiL was low but enough to change C, N, and P concentrations and their relationships. Our study reports for the first time changes in the stoichiometric ratio and respective effects on nutrient use efficiency in Si accumulator plant supplied via foliar spraying. Although foliar spraying of Si has already been evaluated in sorghum plants [18] and PSS without stress [19], the structural effect of this element accumulated in leaf tissue has not yet been observed. Such an effect may have occurred due to spray solution quality, which had low polymerization rates due to the Si concentration used (3.4 mmol L^−1^). In solution, Si can start its polymerization from a concentration of 3.0 mmol L^−1^ [25]. Therefore, at low Si concentrations, Si may maintain its monomeric form (H_4_SiO_4_). Moreover, the humectant properties of the used stabilizer (sorbitol) may have increased of Si monomeric forms and droplet persistence on leaf surface [39], increasing Si absorption per leaf area, mainly during cuticle penetration (passive percolation or surface adsorption) [18].

Another important aspect in our study to maintain the monomeric form of monocyclic acid was acidification at spraying time by reducing spray solution pH (5.5±0.2). Silicon acidified solutions increase concentration of monomeric species [40], which is Si form that reaches cell cytosol [15], improving spray solution quality. Thus, the high efficiency of foliar Si supply enables its use in seedling nurseries, because, despite providing low Si concentration in the leaf tissue, it is sufficient to increase CUE, NUE, PUE and dry matter yield in PSS under no water stress or even short and long-term WD.

The increased amount of Si in leaf tissue altered nutrient concentrations (Figs 2, 5 and 9). Silicon reduced C concentration, hence its incorporation in plant tissues can replace part of the C in cell-wall organic compounds. Energy costs (NADPH and ATP) for Si incorporation into structural compounds is 50 times lower than into organic compounds due to the intrinsic permeability of lipid bilayers [15]. Silicon is immobilized in cell walls in the form of phytoliths, which are structural materials made of a combination of biomolecules similar to lignin [41]. Thus, lignin demand is reduced when plants accumulate Si, suggesting an induced competitive advantage since, plants spend less energy with costly metabolisms of compounds such as in lignin and cellulose synthesis [14, 41] or defense metabolism to stresses (e.g., water deficit). This indicates that Si can improve lignocellulosic biomass for bioenergy production, as lignin is a physical barrier to cellulase action in the enzymatic saccharification process [42].

Another probable Si mechanism to decrease C concentration of C is to decrease transpiration rate, which causes the stomata to close for longer periods during the day [21, 43]. This, in turn, leads to the decrease in water loss in plants under short and long-term WD, with Si supply in both forms of application (Figs 6A and 10A), thus maintaining C assimilation in low water availability. This scenario with higher water content in plants decreased oxidative stress by reducing damage caused by reactive oxygen species, which degrade organic compounds such as membrane lipids, causing cytosol efflux [44]. Such efflux is measured by electrolyte leakage index of leaf cells, which increased in plants with -Si under short and long-term WD (Figs 6B and 10B).

The C concentrations decline in leaf tissue by has also been reported in wheat plants [13] and *Phragmites australis* [14] without water deficit, and in sorghum plants grown in saline solution [45]. Given our results, it also occurs in sugarcane seedlings under suitable conditions and short and long-term WDs. Thus, both SiR and SiL can increase tolerance to WD in sugarcane, as it allows the plant to save energy to cope with stress.

The partial control of C flow by Si changed N and P concentrations. Silicon supply in SiL increased N concentration in plants under prolonged WD (Fig 9B). However, it was reduced with application in SiR and under short-term WD (Fig 5B). This suggests that the time of exposure to stress interferes with Si-induced structural effect. Silicon supply in SiR in wheat plants without water deficit has negative correlation between N and Si concentrations in the leaf tissues [42]. These results demonstrate that leaf spraying is more efficient in increasing N concentration and that prolonged WD intensifies such increase.

Silicon also increased P concentration in plants under short-term WD (Fig 5C), which can be explained by the high stock of inorganic P in the cell vacuole [13]. That was also reported for rice plants [17] and sugarcane [21] with Si addition in nutrient solution without water deficit. On the other hand, prolonged severe WD (30% WRC) decreased P concentration in PSS of sugarcane (Fig 9C), but Si supply contributed to plant development since PUE increased, ensuring greater production of dry matter. This shows that Si supply and stress level interfere with P metabolism, with consequent effects on nutrient stoichiometry and use efficiency.

Changes in the concentration of nutrients interfered with their proportions. Elementary relationships between nutrients are especially important because the concentration of an element in plant tissues is affected by biomass production. This, in turn, interferes with ecological interactions, since these nutrients are strongly related to the biochemical and physiological functioning of plants [12]. The C: N ratio increased with Si supply in unstressed plants of the cultivar CTC 4 (Experiment I) (Fig 2D) and decreased only in plants under long-term WD and Si fertilization via SiL (Experiment III) (Fig 9D). This opposite effect is due to WD because, although Si induced a decrease in C concentrations in both water conditions, only in long-term WD such decrease was followed by N concentration increase.

The decreasing effect of in Si on C concentration, associated with a P concentration raise, decreased the C: P ratio in plants with and without WD (Figs 2E, 5E and 9E). Yet, the N: P ratio did not follow the same pattern for Si supply forms and stress conditions (Figs 2F, 5F and 9F). The lowest C: N and C: P ratios indicate that Si contributes to maintaining the balance between C and the other elements (N and P), even in plants under water deficiency. Plants can adjust their nutrient demands to maintain homeostasis [12]. The increase in Si absorption, mainly in plants under stress, contributes to maintain homeostasis and balance nutrient ratios in leaf tissue, increasing the amount of C-rich structural components, with metabolic function at the expense of structural function.

Physiological responses, such as high photosynthetic efficiency due to improved CO_2_ use by a greater amount of photosynthetic pigments such as chlorophylls [43]_,_ can be among the C metabolic responses in plants supplied with Si when under WD. This was evidenced in plants under short and long-term WDs in our study (Figs 6C and 10C). Higher Chl a+b in plants fertilized with Si under WD have also been reported in sugarcane plants [8]. Thus, Si optimizes energy metabolism and enhance plants drought resistance, with responses varying with WD level and duration.

Beneficial effects in alleviating water stress damage to PSS have been previously demonstrated [11]. However, these authors did not relate plant morphological and physiological gains to changes in C: N: P stoichiometry. Our research shows that for a better understanding of WD effects on sugarcane growth and C: N: P stoichiometric ratios, the losses in CUE, NUE, and PUE should be considered rather than only its physiological damages. PSS of sugarcane submitted to WD stress has a reduced CUE, which hindered biomass production (Figs 7 and 11). This is because the redistribution of nutrients in plants under WD reduces, which can impair their use efficiencies [46]. In turn, Si supplied in SiR or SiL increased CUE in plants, whether stressed or unstressed. This can be attributed to a greater Si incorporation in plant tissues, with its use in photosynthetically rather than in structural compounds [14], as C is replaced by Si [15].

Optimization of Si-induced C metabolism improves C-skeletons production and metabolism of structural nutrients (e.g., N and P). These constitute organic compounds vital to plant, as they are strongly associated with plant biochemical and physiological processes [12]. The improvement of N-use efficiency by Si supply may have resulted from changes in primary metabolism, stimulating translocation of amino acids from source to sink tissues [47, 48]. Silicon supply can also stimulate photosynthetic rate by improving carbonic anhydrase activity, as well as increasing mesophyll conductivity and CO_2_ flow from mesophyll intercellular air spaces to fixation sites in the stroma of chloroplasts of the vascular bundle sheath cells [47]. Such an increased NUE by Si has already been reported in winter wheat, showing biomass production increased [13].

Furthermore, the better CUE and NUE suggests that an energy cost reduction in Si incorporation by substitution of organic compounds has consequently increased PUE. Positive correlations between Si and P concentrations in plant tissue have been observed previously by Schaller et al. [14]. These findings allow us to accept the hypothesis that Si increases CUE, as well as NUE and PUE, which are expressed as biomass produced per unit of nutrient accumulated. Thus, Si fertilization during the formation of PSS of sugarcane can enhance P and N mineral fertilization responses and reduce WD damage at initial growth stage.

The above-described nutrient use efficiency improvements may also plant height increases (Figs 6D and 10D) and dry matter gain in PSS of sugarcane. Also, Si supply via roots during seedling formation was more effective to increase dry matter yield under short-term stress (Experiment II), just as under longer-lasting stress conditions (Experiment III). Our results thus add another Si beneficial effect on sugarcane seedlings since it reduces short- and long-term WD damages by changing stoichiometric relationships, increasing C, N, and P nutritional efficiency and biomass accumulation. In short, our finding help explain the major of this element for sugarcane crop, already stated by Li et al. [49].

## 5. Conclusion

Silicon foliar spraying promotes structural effects even on plants with an active absorption mechanism such as sugarcane, regardless of the water condition. Either root or foliar silicon applications change sugarcane elementary C: N: P stoichiometry and increase the carbon use efficiency, which contributes to increasing nitrogen and phosphorus use efficiencies. This greater efficiency in nutrients use contributes to the adjustment of physiological parameters and dry matter increase in pre-sprouted sugarcane seedlings, both under water deficit short-term induced by PEG as well long-term in soil or suitable water condition. Therefore, the effect os silicon on nutritional carbon efficiency demonstrated in this study should encourage new lines of research on silicon relationship with the water deficit in other plant species.

## Supporting information

S1 Fig Linear regressions between Si and C, N, P in plants of Experiment I. (TIF)

S2 Fig Linear regressions between Si and C, N, P in plants of Experiment II. (TIF)

S3 Fig Linear regressions between Si and C, N, P in plants of Experiment III. (TIF)

S1 File Supporting information data. This file contains all data the plants of experiment I.
(XLSX)

S2 File Supporting information data. This file contains all data the plants of experiment II.
(XLSX)

S3 File Supporting information data. This file contains all data the plants of experiment III.
(XLSX)
