# Supplementary figures and images for: Root- and foliar-applied silicon modifies C: N: P ratio and increases the nutritional efficiency of pre-sprouted sugarcane seedlings under water deficit
Source: PLoS One. 2020 Oct 15;15(10):e0240847. doi: 10.1371/journal.pone.0240847 (PMC7561170; doi:10.1371/journal.pone.0240847)

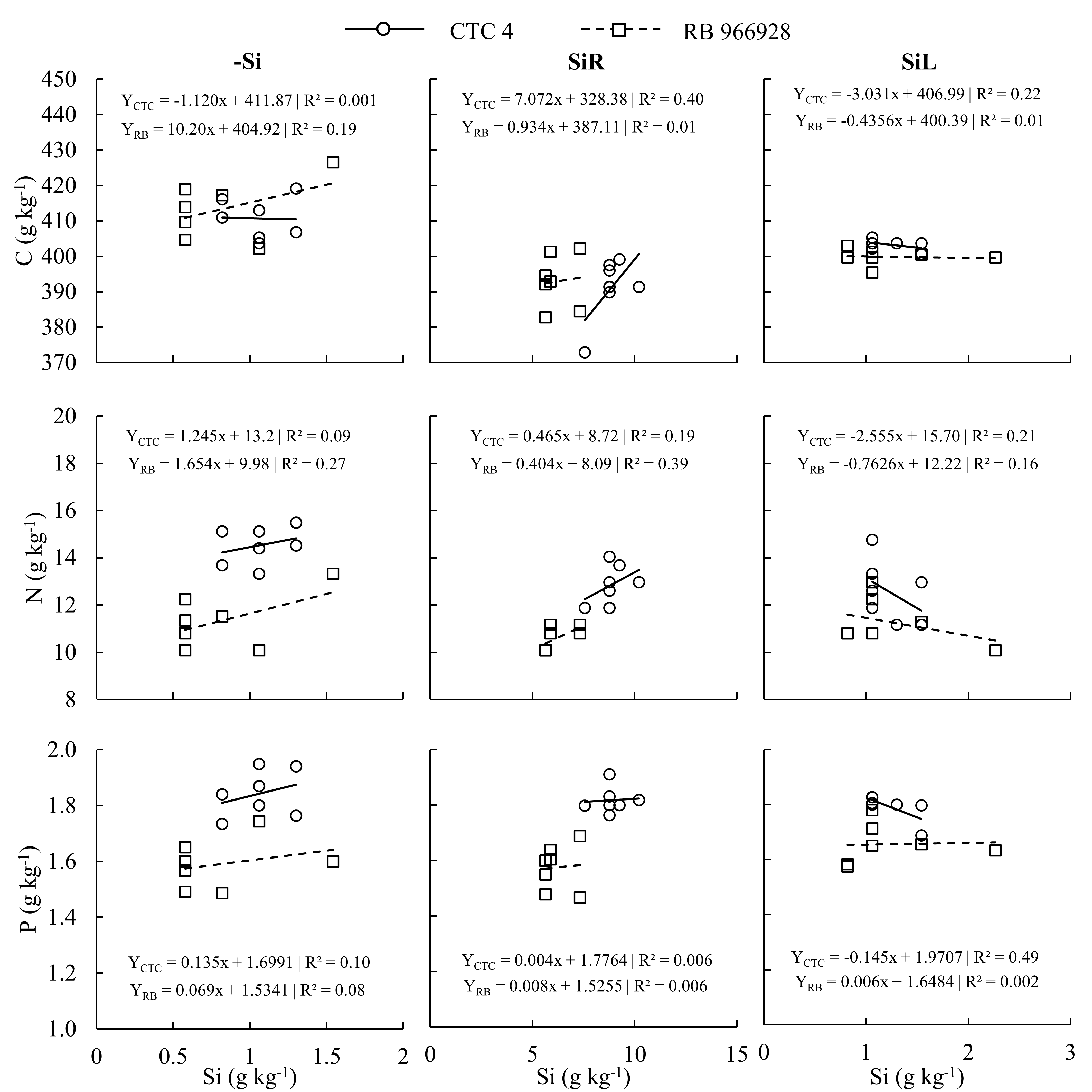

Supplement: S1 Fig — (TIF) [file pone.0240847.s001.tif]

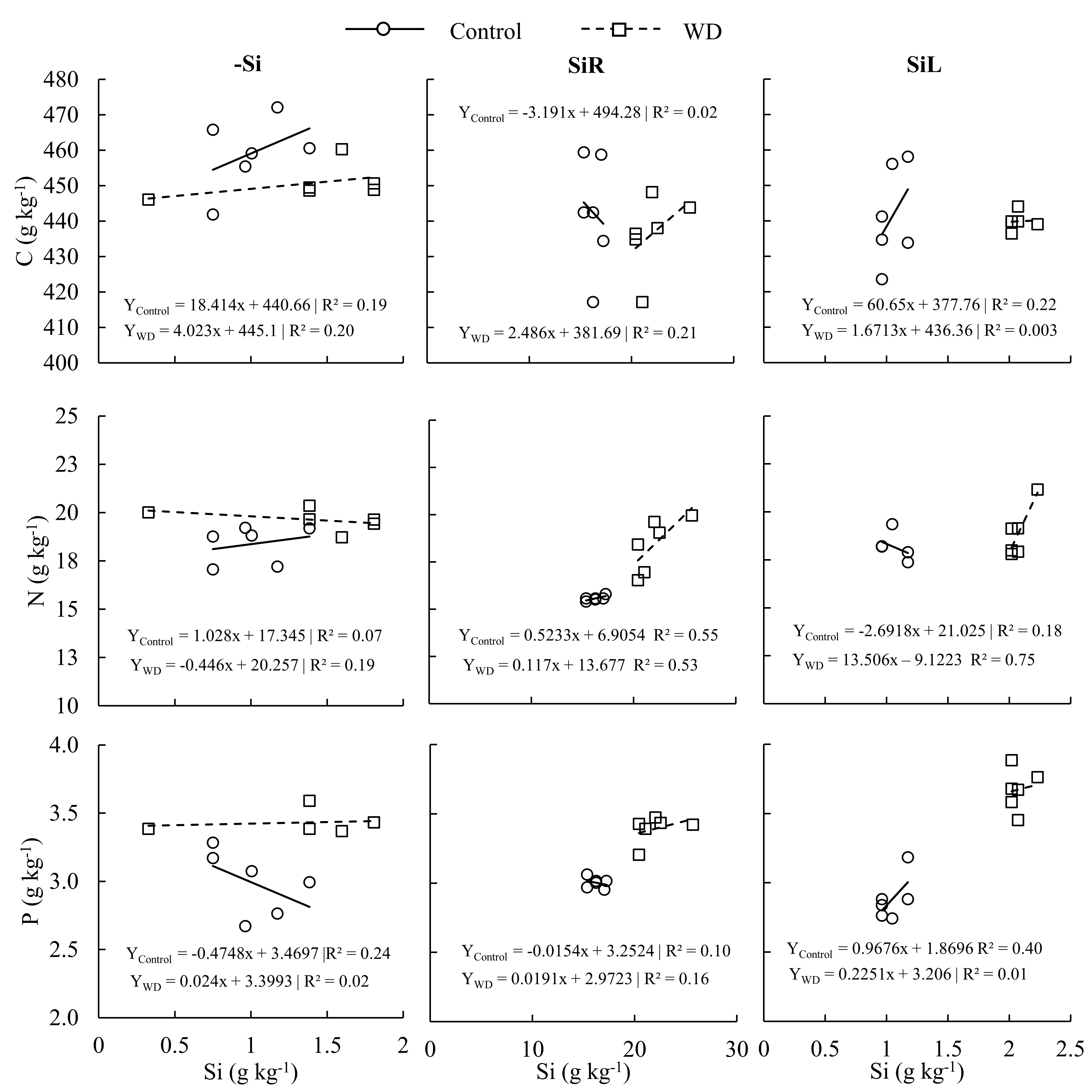

Supplement: S2 Fig — (TIF) [file pone.0240847.s002.tif]

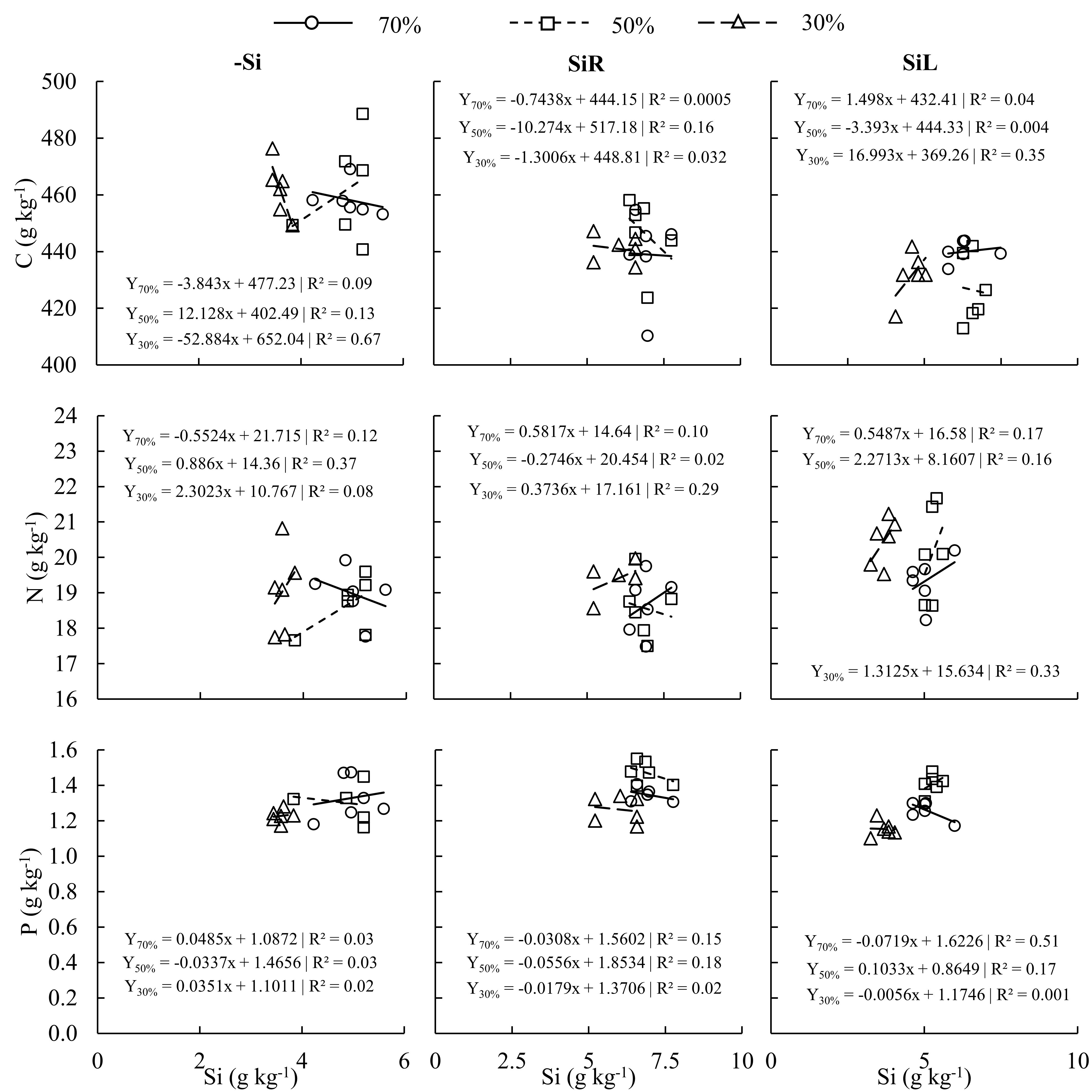

Supplement: S3 Fig — (TIF) [file pone.0240847.s003.tif]
